# Increase of macrolide resistance among *Streptococcus pyogenes* pharyngitis driven by a *mef*(A)-*msr*(D)/*emm*2-ST55 lineage in Portugal (2014–2019)

**DOI:** 10.1128/aac.00968-25

**Published:** 2025-10-10

**Authors:** Ana Friães, Rafael Mamede, Beatriz Santos, Gina Marrão, José Melo-Cristino, Mario Ramirez

**Affiliations:** 1 Instituto de Microbiologia, Faculdade de Medicina,Universidade de Lisboa 37809, Lisbon, Portugal; 2 Gulbenkian Institute for Molecular Medicine 705996, Lisbon, Portugal; 3 Serviço de Patologia Clínica, Laboratório de Microbiologia, Unidade Local de Saúde da Região de Leiria 679532 , Leiria, Portugal; The Peter Doherty Institute for Infection and Immunity, Melbourne, Victoria, Australia

**Keywords:** whole genome sequencing, molecular typing, antimicrobial resistance, streptococcal pharyngitis, Group A Streptococcus

## Abstract

Increases in macrolide resistance occurred recently among *Streptococcus pyogenes* (Group A *Streptococcus*, GAS) in some countries. While the importance of monitoring the clinical and molecular epidemiology of non-invasive GAS is increasingly recognized, most surveillance focuses on invasive infections, since culture is rarely performed in tonsillo-pharyngitis. We determined the antimicrobial susceptibility and characterized the macrolide-resistant lineages of 2,002 pharyngeal isolates recovered in a Portuguese hospital in 2014–2019. There were seasonal variations in the numbers of recovered isolates, with peaks shifting between March–July and October–December, but consistently low numbers in August and September. Macrolide-resistant and macrolide-susceptible GAS presented independent seasonal and clonal dynamics, with resistant isolates showing lower genetic diversity and minimal overlap with susceptible lineages. Overall, 84 (4%), 77 (4%), and 52 (3%) isolates were resistant to erythromycin, clindamycin, and tetracycline, respectively. Until 2018, macrolide resistance was mainly due to an internationally disseminated *emm*77-ST63 lineage carrying *erm*(A) and *tet*(O) in ICE*Sp2905* and an *emm*75-ST49 lineage carrying *mef*(A)-*msr*(D) in a novel ɸ1207.3 variant. In 2019, resistance peaked at 9% due to the rapid expansion of *mef*(A)-*msr*(D)-positive *emm*2-ST55 isolates, replacing previous lineages. Other minor resistant lineages carried mostly *erm*(B) in a diversity of mobile genetic elements, including *emm*75-ST150, *emm*9-ST75, *emm*11-ST403, *emm*12-ST36, *emm*76-ST50, and *emm*77-ST399 [*erm*(T)]. Tetracycline resistance was associated with the genes *tet*(M) and *tet*(O), in most cases co-located in the same genetic elements as the *erm* genes. This study reveals clonal changes among macrolide-resistant GAS driving fluctuations in macrolide resistance and associated phenotypes.

## INTRODUCTION

*Streptococcus pyogenes* (Lancefield Group A *Streptococcus*, GAS) remains uniformly susceptible to penicillin and other β-lactams, but macrolides and lincosamides offer alternatives for β-lactam-allergic patients and the association of clindamycin with a β-lactam is recommended for the treatment of severe invasive GAS infections (1). However, macrolide and lincosamide resistance among GAS presents marked geographical and temporal variations that have been associated with both antibiotic consumption and clonal dynamics (1, 2). Recently, an increase in macrolide resistance among invasive GAS was reported in the United States (3), and the World Health Organization included macrolide-resistant GAS in its Bacterial Priority Pathogens List in 2024 (4).

Macrolide resistance in GAS is mostly associated with the presence of the *erm* family of genes or the *mef*(A/E)-*msr*(D) operon. The *erm* genes confer cross-resistance to macrolides, lincosamides, and streptogramin B, either constitutive (cMLS_B_ phenotype) or induced by a macrolide (iMLS_B_ phenotype), due to rRNA methylation (1, 2). Carriage of *mef*(A/E)-*msr*(D) results in resistance to macrolides alone (M phenotype) by a mechanism likely involving ribosomal protection in addition to active efflux (5, 6). The *erm* and *mef*(A/E)-*msr*(D) genes are carried on a variety of mobile genetic elements (MGEs), which allow the horizontal dissemination of macrolide resistance while frequently carrying other resistance determinants (2).

Macrolide resistance among pharyngeal isolates recovered throughout Portugal declined from 28% in 2000 to 1% in 2013 (7). This decrease was not associated with marked changes in macrolide consumption but was instead accompanied by multiple clonal changes (7–9). Since 2013, national guidelines discourage the use of culture for the etiological diagnosis of tonsillopharyngitis in favor of rapid antigen detection tests (10). Several European countries follow similar practices hampering the monitoring of antimicrobial resistance among pharyngeal GAS. Although macrolide resistance among invasive GAS in Portugal remained below 5% throughout the 2010s (11), we had previously found a significantly higher macrolide resistance among pharyngeal isolates than contemporaneous invasive isolates, highlighting the potential of the pharynx as a reservoir of macrolide-resistant GAS (12).

To gain a more comprehensive view of the clonal landscape of macrolide-resistant GAS after 2013, we determined the antimicrobial susceptibility and characterized genomically a collection of pharyngeal GAS isolates prospectively collected in a Portuguese hospital center in 2014–2019.

## MATERIALS AND METHODS

### Bacterial strains

Between January 2014 and December 2019, pharyngeal swabs were obtained and cultured from suspected cases of streptococcal tonsillo-pharyngitis at Unidade Local de Saúde da Região de Leiria, Leiria, Portugal (ULSRL). ULSRL includes three tertiary hospitals covering an estimated population of 400,000 inhabitants in the center region of Portugal. GAS isolates were identified by colony morphology, β-hemolysis, and detection of the Lancefield group A antigen. The Institutional Review Board of Centro Académico de Medicina de Lisboa approved the study (258/22–AD/24). The study was exempt from obtaining written informed consent from the patients because it used only anonymized demographic data and involved samples collected as part of routine diagnostic procedures, with no additional intervention or identifiable information.

### Antimicrobial susceptibility testing

Susceptibility testing was performed by disk diffusion on Mueller-Hinton agar supplemented with 5% defibrinated sheep blood, according to the guidelines and interpretative criteria of the Clinical and Laboratory Standards Institute (CLSI) (13), using the following disks (Oxoid, Basingstoke, UK): penicillin, erythromycin, clindamycin, tetracycline, and levofloxacin. The D-zone test was used to detect inducible clindamycin resistance (13).

### Whole genome sequencing

A total of 391 pharyngeal GAS isolates were subjected to whole genome sequencing (WGS) as previously described (14), including all erythromycin-resistant isolates recovered at ULSRL during 2014–2019 (*n* = 84), one-third of the erythromycin-susceptible isolates recovered at ULSRL during 2017–2019, selected randomly (*n* = 287), and erythromycin-resistant isolates recovered throughout Portugal during 1998–2013, previously characterized (7–9) and belonging to *emm*2, *emm*75, and *emm*77 (*n* = 20) (Table S1). *De novo* assembly and determination of *emm* types, seven-gene multilocus sequence types (STs), core-genome multilocus sequence typing (cgMLST) profiles, and antimicrobial resistance genes were performed as described elsewhere (14, 15). The genomic analyses also included 215 genetically, temporally, geographically, and clinically diverse isolates of *emm*2, *emm*11, *emm*12, *emm*75, and *emm*77 selected from the Davies et al. data set (16) according to previously detailed criteria (14), as well as eight *emm*77-ST63 isolates from Poland (17) (Table S2). MGE identification relied on BLAST searches and alignments with public sequences of known MGEs carrying macrolide resistance determinants, using ICEfinder (18) to identify putative integrative conjugative elements (ICEs). Detailed methods for WGS and data analysis are available as supplemental material.

### Statistical analysis

Isolate diversity was evaluated using the Simpson’s Index of Diversity (SID) with corresponding 95% confidence interval (CI_95%_) (19). Two-tailed Fisher’s exact test and odds ratios were used to identify significant pairwise associations. The Cochran-Armitage test was used to evaluate trends. The *P*-values for multiple tests were corrected with the false discovery rate (FDR) linear procedure (20). Values of *P* < 0.05 were considered significant.

## RESULTS

### Isolate distribution and demographic data

A total of 2,002 non-duplicate GAS isolates were cultured from pharyngeal swabs of patients with tonsillo-pharyngitis at ULSRL in 2014–2019 with an average of 334 isolates per year (range 264–401). Only four patients were adults (≥18 years old), and the median age was 6 years (interquartile range 4–9 years). Fifty-two percent of the patients were female. Age and sex were unknown for one patient.

**Fig 1:**
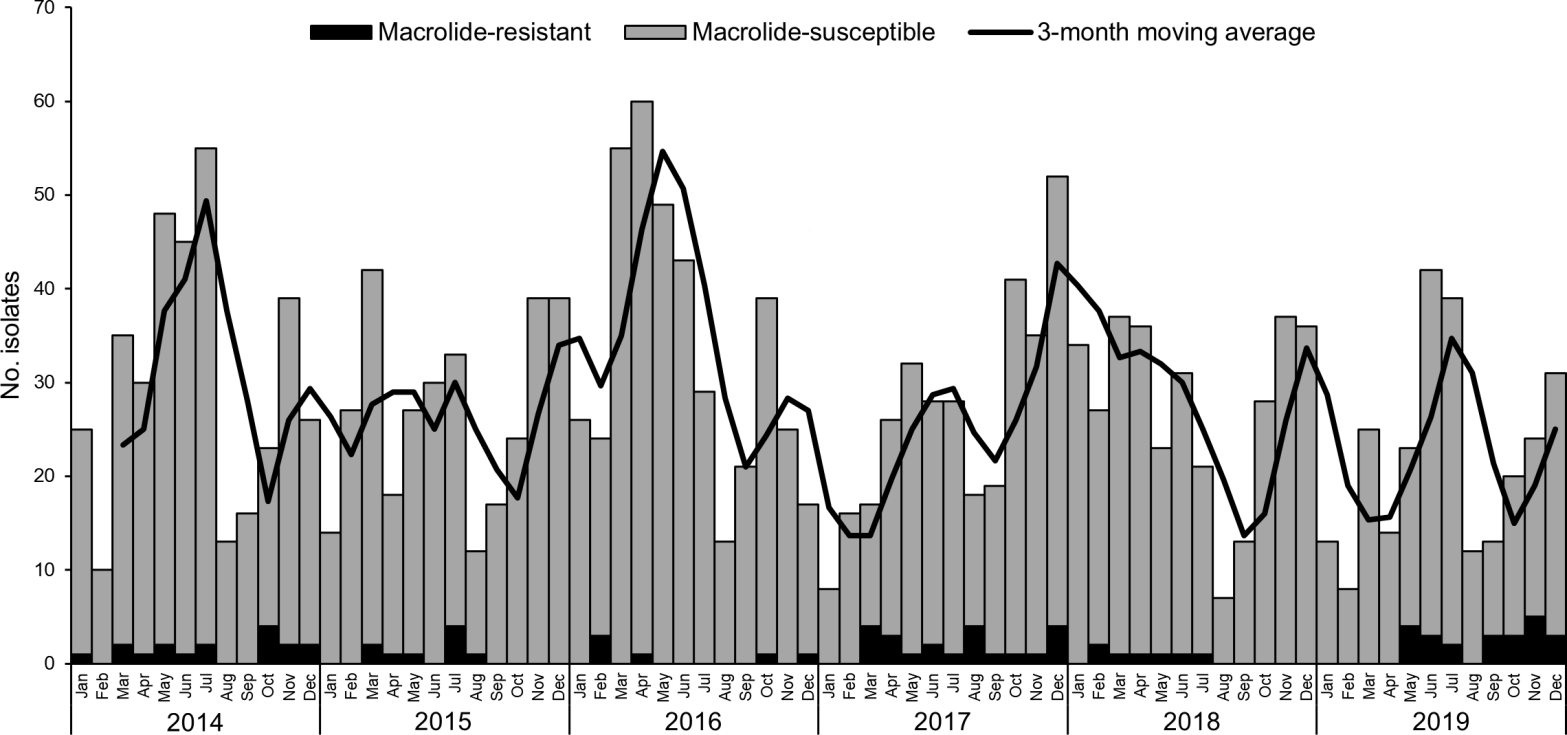
Monthly distribution of 2,002 pharyngeal GAS isolated between 1 January 2014 and 31 December 2019. The black line represents the 3-month moving average of the total isolates.

The isolates were not evenly distributed throughout the year, and differences were seen between years (Fig. 1). The number of isolates was consistently low during August–September, and in most years, January and/or February also presented low isolate counts. In general, there were two peaks each year, one during Spring/early Summer (varying between March and July) and the other in Autumn, between October and December. The distribution of macrolide-resistant isolates was different from that of macrolide-susceptible isolates, with no clear seasonal pattern.

### Antimicrobial resistance

All isolates were susceptible to penicillin and levofloxacin. Erythromycin presented the highest resistance rate (*n* = 84, 4.2%), followed by tetracycline (*n* = 77, 3.8%, of which 51 isolates were also erythromycin-resistant), and clindamycin (*n* = 52, 2.6%).

**Fig 2:**
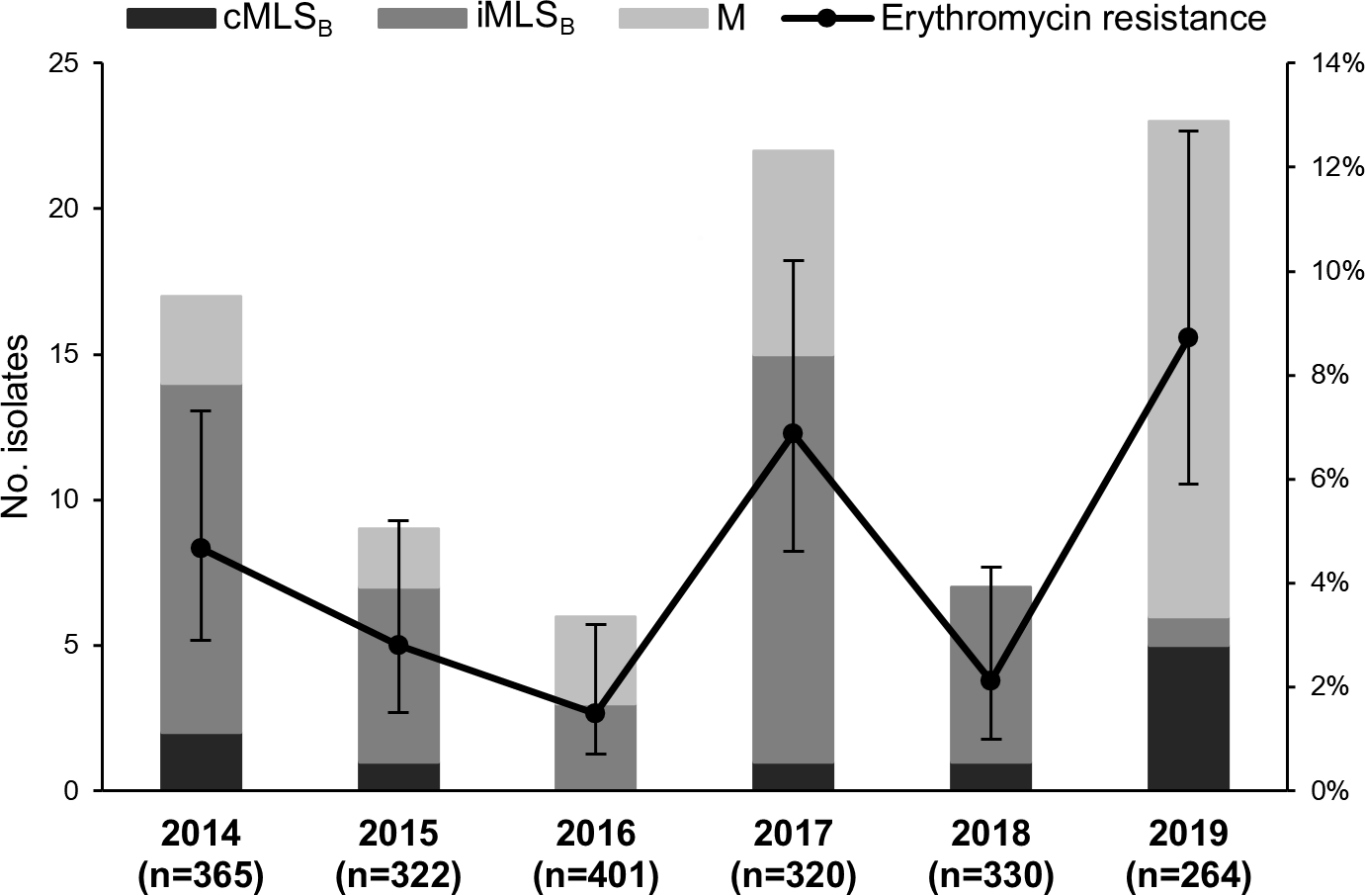
Yearly distribution of erythromycin resistance and associated phenotypes among 2,002 pharyngeal GAS isolated between 1 January 2014 and 31 December 2019. Error bars represent 95% CIs. cMLS_B_, constitutive resistance to macrolides, lincosamides, and streptogramin B; iMLS_B_, inducible resistance to macrolides, lincosamides, and streptogramin B; M, resistance to macrolides and susceptibility to lincosamides and streptogramin B.

Erythromycin resistance remained mostly <5%, decreasing between 2014 and 2016 (*P* = 0.01). However, there were two peaks, one of 6.9% in 2017 and another of 8.7% in 2019 (Fig. 2). Up to 2018, the dominant macrolide resistance phenotype was iMLS_B_. After a complete suppression in 2018, the M phenotype emerged as the dominant phenotype in 2019, accompanied by an increase in cMLS_B_, while iMLS_B_ was found in only one isolate.

### Comparison between macrolide-resistant and macrolide-susceptible isolates

**Fig 3:**
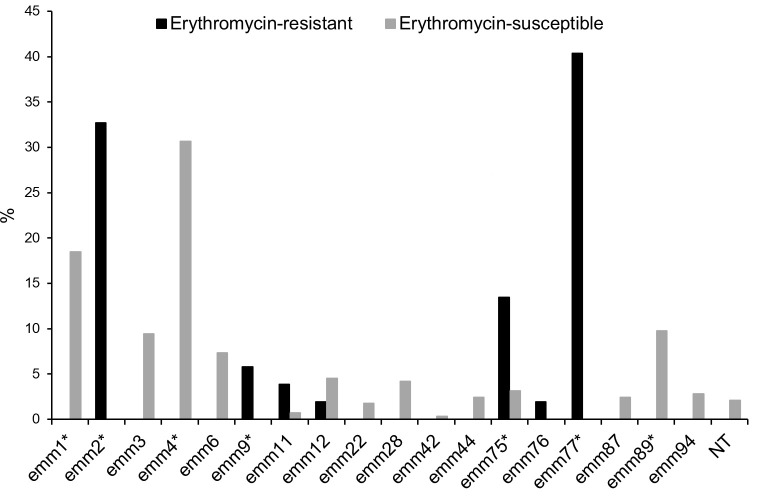
*emm* type prevalence among erythromycin-resistant (*n* = 52) and macrolide-susceptible (*n* = 287) pharyngeal GAS isolated between 1 January 2017 and 31 December 2019. NT, non-typable due to *emm* gene deletions or fusions; ^*^P < 0.05, significant after FDR correction.

In order to compare the clonal composition of macrolide-resistant and susceptible GAS causing pharyngitis, one-third of the susceptible isolates from each year between 2017 and 2019 were randomly selected for WGS. During this period, resistant isolates (*n* = 52) presented a significantly lower diversity of *emm* types (seven *emm* types, SID = 0.720 [CI_95%_ 0.644–0.797]) and STs (8 STs, SID = 0.735 [CI_95%_ 0.660–0.811]) than susceptible isolates (*n* = 287; 14 *emm* types, SID = 0.837 [CI_95%_ 0.810–0.864]); 25 STs, SID = 0.860 [CI_95%_ 0.832–0.887]) (*P* < 0.01). Furthermore, the *emm* type distribution differed markedly between the two subgroups, with four *emm* types overrepresented among macrolide-resistant isolates (*emm*2, *emm*9, *emm*75, and *emm*77), and three *emm* types associated with the macrolide-susceptible subset (*emm*1, *emm*4, and *emm*89) (Fig. 3). Only *emm*11, *emm*12, and *emm*75 co-occurred among macrolide-susceptible and macrolide-resistant isolates.

### Genetic lineages associated with macrolide resistance

The most common genetic lineage among macrolide-resistant isolates, identified in all study years, was an *emm*77-ST63 lineage (*n* = 41, 49%), carrying *erm*(A) and *tet*(O) and presenting the iMLS_B_ phenotype and tetracycline resistance (Table 1). The minimum-spanning tree (MST) of the cgMLST profiles of these isolates was constructed together with those of other *emm*77 isolates, including pharyngeal macrolide-resistant isolates recovered previously throughout Portugal (*n* = 9) (7), isolates from Poland (*n* = 8) (17), and genetically diverse isolates from multiple countries (*n* = 41) (16) (Tables S1 and S2). The MST shows four main genetic lineages and one singleton separated by >1,000 allelic differences (from a total of 1,314 compared loci) (Fig. S1). All *emm*77-ST63 isolates from Portugal were clustered in the same lineage, together with isolates from multiple continents presenting either ST63 or its single-locus variant (SLV), ST1023. Regardless of isolation date and hospital, isolates from Portugal were closely related, with most MST links ranging between 0 and 11 allelic differences (the exceptions being two isolates from 2007 to 2013 linking at 23 allelic differences). Isolates from the United States (*n* = 2) and the United Kingdom (*n* = 2) carrying *erm*(A), as well as one isolate from Australia with no detected macrolide resistance genes, were also closely grouped to isolates recovered in Portugal during 2007–2013 (MST links of 14–24 allelic differences). The single isolate in our collection harboring *erm*(T) also belonged to *emm*77 but presented an unrelated ST (ST399) and grouped into a distinct lineage, close (five allelic differences) to an ST399 isolate from the United States that also carried *erm*(T).

**TABLE 1:** Characteristics of the macrolide-resistant isolates (*n* = 84) identified among 2,002 pharyngeal GAS isolated between 1 January 2014 and 31 December 2019^*a*^

*emm* type	ST	Resistance phenotype	Resistance genes*^b^*	No. isolates						
				2014	2015	2016	2017	2018	2019	Total
*emm*77	63	iMLS_B_, Tet	*erm*(A), *tet*(O)	12	6	3	14	5	1	41
	399	iMLS_B_, Tet	*erm*(T), *tet*(M)					1		1
*emm*2	55	M	*mef*(A)-*msr*(D)						17	17
*emm*75	49	M	*mef*(A)-*msr*(D)	3	2	3	7			15
	150	cMLS_B_, Tet	*erm*(B), *tet*(M)	1	1					2
*emm*9	75	cMLS_B_, Tet	*erm*(B), *tet*(M)						3	3
*emm*11	403	cMLS_B_, Tet	*erm*(B), *tet*(M)						2	2
		cMLS_B_	*erm*(B)	1						1
*emm*12	36	cMLS_B_, Tet	*erm*(B), *tet*(M)				1			1
*emm*76	50	cMLS_B_, Tet	*erm*(B), *tet*(M)					1		1

^*a*^ ST, sequence type; cMLS_B_, constitutive resistance to macrolides, lincosamides, and streptogramin B; iMLS_B_, inducible resistance to macrolides, lincosamides and streptogramin B; M, resistance to macrolides and susceptibility to lincosamides and streptogramin B; Tet, resistance to tetracycline.

^*b*^ Only genes conferring resistance to macrolides and tetracycline are presented. Other resistance genes identified with ABRicate are presented in Table S1.

The *emm*2-ST55 lineage, exhibiting the M phenotype and carrying the *mef*(A)-*msr*(D) gene combination, comprised 17 isolates (20%). An MST was constructed with these isolates and all *emm*2 isolates in our collection of pharyngeal isolates recovered during 1998–2013 throughout Portugal (*n* = 5) (7, 9), as well as with the *emm*2 isolates present in the diverse collection from Davies et al. (16) (Fig. S2). Despite having been recovered in six countries from four continents throughout >20 years, the *emm*2 isolates presented a very limited genetic diversity, all belonging to ST55 and with MST links ranging from 0 to 59 allelic differences in a total of 1,427 core loci. The isolates recovered in the present study formed a subcluster with distances of 0–3 allelic differences, in agreement with their close geographic and temporal clustering (all recovered in 2019). Remarkably, isolates from Portugal were the only isolates carrying macrolide resistance genes. However, despite sharing the same resistance genes (*mef*(A)-*msr*(D)), isolates from 1998 to 2013 were more closely related to macrolide-susceptible isolates from several other countries than to the 2019 macrolide-resistant isolates from ULSRL.

The *emm*75-ST49 lineage carrying *mef*(A)-*msr*(D) accounted for all the M phenotype isolates between 2014 and 2017 (*n* = 15, 18%) but completely disappeared in 2018 and 2019 after peaking in 2017 (7/22 isolates, 32%). Using cgMLST data, all *emm*75 isolates from the present study (including 17 macrolide-resistant and nine macrolide-susceptible isolates) were compared with pharyngeal, macrolide-resistant *emm*75 isolates recovered previously throughout Portugal (*n* = 6) (7), and with the *emm*75 assemblies from the Davies et al. data set (*n* = 40) (16). The respective MST reflects a high genetic diversity, presenting three main genetic lineages and a singleton separated by >1,000 allelic differences (in a total of 1,305 core loci), and multiple links > 100 allelic differences (Fig. S3). All *emm*75-ST49 isolates carrying *mef*(A)-*msr*(D) from our study were closely clustered (MST links of 0–5 allelic differences) and were genetically related (21 allelic differences) to a strain from Canada carrying no macrolide resistance genes. All isolates from the analyzed data set presenting ST49 or its SLV ST861 were grouped in the same sublineage, regardless of carrying the *mef*(A)-*msr*(D) genes or not. The remaining isolates from Portugal were all grouped in a distinct sublineage separated by >489 allelic differences, including the pharyngeal resistant isolates from the national study (2007–2013). The latter, despite sharing the same resistance genes (*mef*(A)-*msr*(D)), presented distinct STs, namely ST150 (double locus variant [DLV] of ST49) or ST657 (SLV of ST150 and triple locus variant [TLV] of ST49). These isolates formed a cluster linked by 43 allelic differences to a strain from Belgium with no macrolide resistance genes. In 2014 and 2015, there were two cMLS_B_ *emm*75-ST150 isolates, also tetracycline-resistant, and carrying the *erm*(B) and the *tet*(M) genes. These were the only two isolates carrying the *erm*(B) gene in the MST and were both grouped close to the macrolide-susceptible isolates recovered in our study during 2017–2019, all sharing ST150.

The MST of *emm*11-ST403 isolates from this study (three macrolide-resistant and two macrolide-susceptible isolates) and the *emm*11 isolates from the Davies et al. data set (*n* = 51) (16) grouped all *emm*11-ST403 isolates into the same sublineage, regardless of geographic origin and of carrying macrolide-resistance genes (Fig. S4).

For *emm*12, we constructed an MST with the isolates from this study (one macrolide-resistant and 13 macrolide-susceptible isolates) together with diverse *emm*12 isolates from Davies et al. (*n* = 69) (Fig. S5). Isolates carrying different macrolide resistance-conferring genes and susceptible isolates were interspersed throughout the MST, regardless of geographic origin.

For the remaining lineages associated with macrolide resistance in our collection (*emm*9-ST75 and *emm*76-ST50), no genomic comparisons were performed, since they were each represented by few isolates (*n* ≤ 3) and were not found among the sequenced macrolide-susceptible isolates.

### Mobile genetic elements encoding macrolide resistance

**Fig 4:**
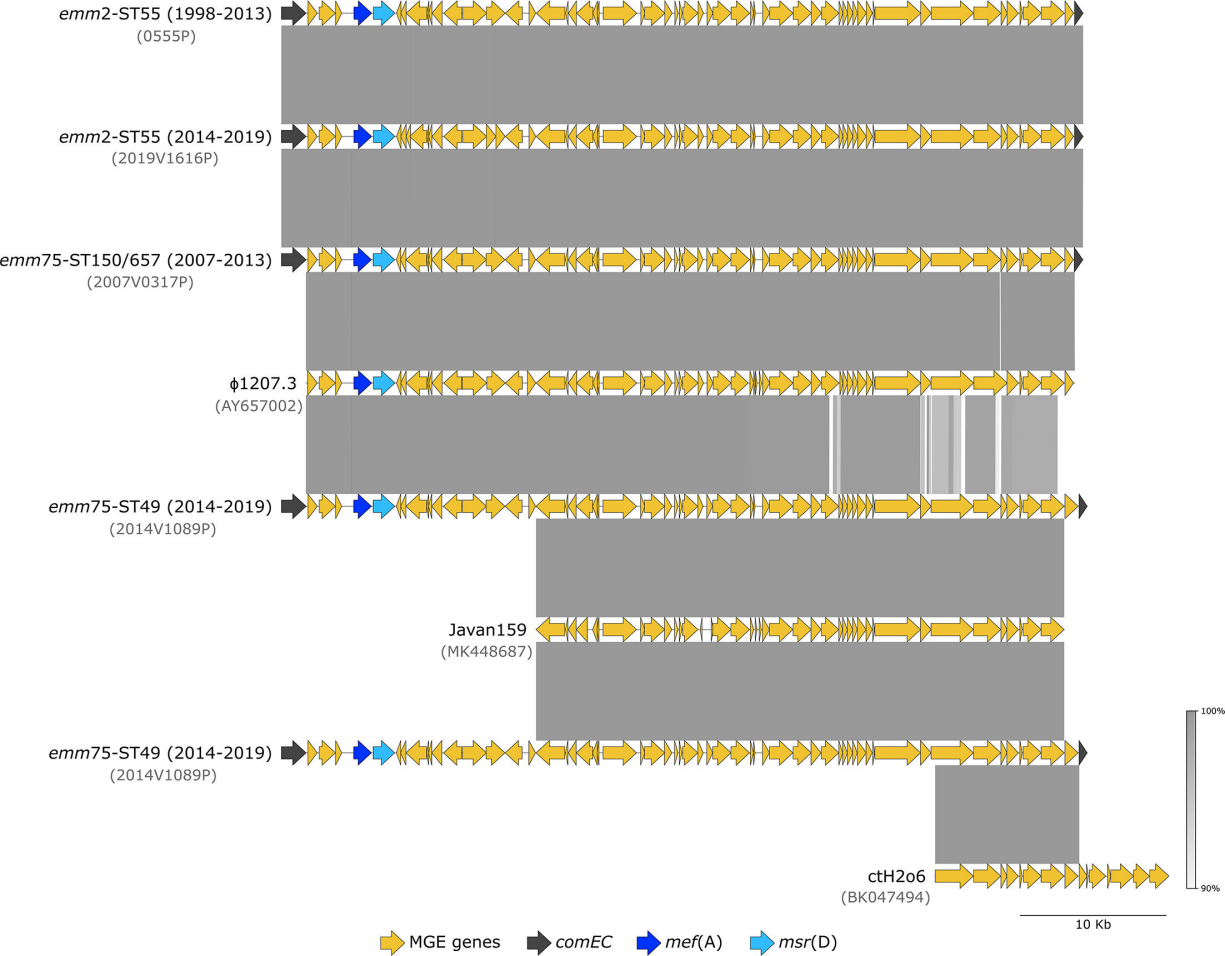
Comparison of the elements carrying *mef*(A)-*msr*(D) genes in *emm*2-ST55, *emm*75-ST150/657, and *emm*75-ST49 isolates from this study, the reference sequence of ɸ1207.3, phage Javan159 from *S. dysgalactiae* subsp. *equisimilis* (24), and the partial sequence of a phage identified by metagenomics in a human sample (ctH2o6) (25). The names of the representative isolates used for each lineage or the GenBank accession numbers of the sequences are indicated in gray. The gray-shaded areas connect regions based on nucleotide identity (90%–100%). Annotated coding sequences are represented as arrows: genes belonging to the MGE in orange, except for *mef*(A) (dark blue) and *msr*(D) (light blue), and the interrupted chromosomal *comEC* gene into which the element is inserted in dark gray.

In M phenotype isolates representing *emm*2-ST55 (from both the 1998–2013 and 2014–2019 periods) and *emm*75-ST150/657 (from 2007 to 2013), the *mef*(A)-*msr*(D) genes were found in elements presenting ≥99.9% nucleotide identity over the entire length of the ɸ1207.3 reference sequence (accession no. AY657002), and inserted into the *comEC* gene as previously described for ɸ1207.3 (21, 22) (Table 2; Fig. 4). In *emm*75-ST49 isolates, the *mef*(A)-*msr*(D)-carrying element inserted into *comEC* was different. It presented 99.9% identity with ɸ1207.3 and other related elements, such as ɸ29862, ɸ29961, and ɸ29854 (accession nos. MT303952, MT311968, and MT311967) (23) but only over the 5′ 35.2 kb sequence. It showed lower identity with any of these elements in the 3′ region (93.8%–93.9% pairwise identity over 17.5 kb). Most of this region was 99.99% identical at the nucleotide level, with phage Javan159 (accession no. MK448687) identified in the draft genome of a *Streptococcus dysgalactiae* subsp. *equisimilis* strain (accession no. ERR111982) (24). However, this prophage lacks the 5′ 15.6 kb region of ɸ1207.3 carrying *mef*(A)-*msr*(D), as well as the 3′-terminal open reading frame (ORF) annotated as encoding a hypothetical protein in the element from our *emm*75-ST49 isolates. This ORF was found in the partial genome of a phage from an unknown host (*Caudoviricetes* sp. isolate ctH2o6, accession no. BK047494) identified in a human sample by metagenomics (25). The 3′-terminal 9.8 kb region of the ɸ1207.3-like element in our *emm*75-ST49 isolates shares 99.97% nucleotide identity with ctH2o6 (Fig. 4). This suggests that the *emm*75-ST49 ɸ1207.3 variant may have emerged through recombination between ɸ1207.3 (or a related element) and other phages.

**TABLE 2:** MGEs carrying macrolide resistance genes among 104 macrolide-resistant pharyngeal GAS sequenced in this study

MGE	Resistance genes	Insertion site	Genetic lineage
ɸ1207.3	*mef*(A)*-msr*(D)	*comEC*	*emm*2-ST55, *emm*75-ST150/657
ɸ1207.3-like	*mef*(A)*-msr*(D)	*comEC*	*emm*75-ST49
ICE*Sp2905*	*erm*(A), *tet*(O)	*rumA*	*emm*77-ST63
Tn*3872*-containing element (putative ICE)	*erm*(B), *tet*(M)	DQL26_RS02190*^a^*	*emm*9-ST75
Tn*3872*-containing element (putative ICE)	*erm*(B), *tet*(M)	*hsdM*	*emm*75-ST150
Tn*3872*-like-containing element (putative ICE)	*erm*(B), *tet*(M)	*hsdM*	*emm*76-ST50
Tn*3872*-like-containing element (putative ICE)	*erm*(B), *tet*(M)	acetate CoA-transferase α subunit gene	*emm*12-ST36
Tn*6003* containing element	*erm*(B), *tet*(M), *aph(3')-IIIa*, *sat4*	*rumA*	*emm*11-ST403
ICE-HKU372-like	*erm*(B)	*hsdM*	*emm*11-ST403
pRW35	*erm*(T)	-	*emm*77-ST399

^*a*^ Hypothetical protein CDS annotated in the genome of strain NCTC8316 (accession no. NZ_LS483521).

The *erm*(A) gene was found exclusively in *emm*77-ST63 isolates (from this study and from the national study of 2007–2013), together with *tet*(O) in an element sharing 99.78% nucleotide identity with the ICE*Sp2905* reference sequence (accession no. FR691055) and 99.98% with the ICE*Sp2905* from *emm*77-ST63 strain 1851/03 from Poland (accession no. NZ_CP155740), inserted into the *rumA* gene (17, 21) (Fig. S6).

The *erm*(B) gene was associated with multiple elements. In *emm*9-ST75, *emm*12-ST36, *emm*75-ST150, and *emm*76-ST50 isolates, *erm*(B) and *tet*(M) were found in Tn*3872*-like elements inserted into larger MGEs identified as putative ICEs in ICEfinder (Table 2; Fig. S7). The element in *emm*9-ST75 isolates appears to be the same element present in Houston isolate TSPY153 (accession no. CP060639), but with a deletion of 1,221 bp resulting in a chimeric coding sequence (CDS). The element is inserted immediately upstream of a hypothetical protein CDS (locus tag DQL26_RS02190) annotated in the complete genome of an *emm*9-ST75 isolate lacking the Tn*3872* element (NCTC8316, accession no. NZ_LS483521). In *emm*75-ST150 isolates, the Tn*3872*-carrying element was inserted into the *hsdM* gene, similarly to isolates of the same lineage recovered in Spain (21). The *emm*76-ST50 isolate presents a Tn*3872*-like element with a transposase inserted downstream of *tet*(M), carried in a longer element, which is inserted into the *hsdM* gene. The element found in the *emm*12-ST36 isolate shares 99.99% nucleotide identity with the element carrying *erm*(B) and *tet*(M) in the complete genome of an *emm*12-ST36 strain from Italy (Isolate 26, accession no. CP167006) (26), although it is inserted into a different gene (acetate CoA-transferase α subunit in our isolate and phosphatidylglycerophosphatase A in CP167006). The Tn*3872*-like element in the *emm*12-ST36 isolates shares the lowest nucleotide identity with the reference sequence of Tn*3872* of *Streptococcus agalactiae* (accession no. OP715845) (92.5%) among the Tn*3872*-like elements identified in this study (Fig. S7).

Besides *erm*(B) and *tet*(M), the *emm*11-ST403 isolates from 2019 (*n* = 2) carry a truncated *aadE* gene, as well as the *aph(3')-IIIa* and *sat4* genes encoding aminoglycoside and streptothricin modifying enzymes, suggestive of a Tn*6003*-like element (27). Although the complete element was not assembled into a single contig in our isolates, read mapping supported the presence of Tn*6003*-like elements similar to those found in the complete genome of an *emm*11-ST403 strain from Italy (Isolate 18, accession no. CP167011) (26). In contrast, the *emm*11-ST403 isolate from 2014 carried only the *erm*(B) gene in an element similar to ICE-HKU372 (28). As in the *emm*12 isolate from Hong Kong where this element was originally identified (accession no. ERR060322), the ICE-HKU372-like element in our isolate is inserted into the *hsdM* gene but has a 1,428 bp sequence encoding two hypothetical proteins inserted into the gene encoding a Cro/CI family transcriptional regulator (locus ERR060322p_11600) (Fig. S8).

Finally, the *erm*(T) gene was found in a single isolate (*emm*77-ST399), on a 5,044 bp contig sharing 99.9% nucleotide identity with plasmid pRW35 (accession no. EU192194). As reported in *emm*77-ST399 isolates from the United States (29), this isolate also carried the *tet*(M) gene located in a different genetic element.

## DISCUSSION

GAS pharyngitis is generally considered to peak during winter and early spring, favored by increased indoor time and crowding (30, 31). Our data support a consistent decrease in the number of cases during mid- and late-summer (August-September), but infection peaks were more variable, frequently occurring in late spring/early summer, while the coldest months of January and February usually presented lower numbers of isolates. This aligns with a study from the Spanish island of Mallorca, which reported a rise in cases starting in March, peaking in June, and followed by a summer slump (32) potentially highlighting different dynamics in temperate climates.

Our results are consistent with macrolide-resistant GAS having their own dynamics, distinct from that of macrolide-susceptible isolates (33). This was reflected in the absence of a seasonal pattern in the distribution of resistant isolates (Fig. 1), as well as in the distinct clonal profiles of the resistant and susceptible subsets during 2017–2019. Resistant isolates presented a lower genetic diversity as measured by ST and *emm* type, with only three *emm* types common to both subsets (*emm*11, *emm*12, and *emm*75) and several overrepresented in either susceptible or resistant isolates. Fluctuations in resistance rates were underpinned by specific clones underlining the importance of clonal dynamics in macrolide resistance in streptococci (34). There were also yearly variations in the prevalence of some of the susceptible clones in 2017–2019 (Fig. S7). Notably, although *emm*12 was always present throughout this period, macrolide-susceptible and -resistant sublineages did not co-exist in the same year.

In periods overlapping with this study, similarly low erythromycin resistance rates (<10%) were reported in Spain (4%–10%, invasive isolates, 2016–2019), Ireland (5%, invasive and non-invasive isolates, 2012–2015), Houston (4%, pharyngeal isolates, 2013–2017), and Canada (8.5%–10%, invasive isolates, 2016–2019) (35–38). However, resistance rates >10% were recorded in Bulgaria (31%, invasive and non-invasive isolates, 2013–2016), Southern Hungary (11%, invasive and non-invasive isolates, 2008–2017), and Central Greece (16%, pharyngeal isolates, 2011–2017) (39–41). These illustrate important geographic variations even within the European region. Notable increases in macrolide resistance were observed in Barcelona, Spain, where resistance increased from 6% in 2013–2015 to 12% in 2016–2018 among invasive and non-invasive infections in adults (21), and in the United States, where resistance among invasive isolates increased from 18% in 2015–2017 to 25% in 2018–2019 mainly due to the expansion of *emm*92 isolates carrying *erm*(T) (3).

In line with pharyngeal isolates collected nationwide during 2007–2013 and with invasive isolates throughout the 2010s (7, 11), overall macrolide resistance among the isolates recovered at ULSRL during 2014–2019 was low (4%), presenting a decreasing trend between 2014 and 2016. In 2017, there was a resistance peak of 7% associated with an increase in lineages that were already circulating in previous years, namely *emm*77-ST63 carrying *erm*(A) and *tet*(O) and *emm*75-ST49 harboring *mef*(A)-*msr*(D). These two lineages sharply decreased (*emm*77-ST63) or completely disappeared (*emm*75-ST49) in 2018–2019, while an *emm*2-ST55 clone carrying *mef*(A)-*msr*(D) surged in 2019, leading to a macrolide resistance peak of 9% and to an inversion in the dominant resistance phenotypes. Similar changes in resistance phenotypes driven by clonal replacements have been previously observed at the national level in GAS (9). Throughout this study, tetracycline resistance was equally low (4%), and 66% of the tetracycline-resistant isolates were also resistant to erythromycin and clindamycin, reflecting the frequent co-occurrence of *tet* and *erm* genes in the same MGEs.

WGS data were used to identify the MGEs carrying macrolide resistance genes, as well as to compare the major genetic lineages found in this study with those previously identified nationwide, and to contextualize them among the international landscape of GAS clones. The dominant *emm*77-ST63 lineage in this study carried an ICE*Sp2509* element and was already circulating nationally in 2007–2013 (7). Isolates of *emm*77-ST63 closely related to ours (Fig. S1) or carrying the same MGE have been identified in other countries, such as the United States, United Kingdom, Poland, and Spain (16, 17, 21). Furthermore, *emm*77 was among the top 10 *emm* types presenting macrolide resistance worldwide during 2000–2020 (42).

In contrast to *emm*77, the *emm*75 isolates carrying *mef*(A)-*msr*(D) in this study represent a distinct genetic sublineage from those recovered during 2007–2013, which was reflected by their separate clustering in the MST (Fig. S3), but also on their different STs (ST49 in the current study and ST150/657 in 2007-2013). Isolates of *emm*75 are known to be genetically diverse and multiple lineages carrying macrolide resistance determinants in different MGEs are geographically spread (16, 21, 42). The *emm*75-ST49 sublineage had not been previously identified in Portugal but was found in the United States, Canada, and Spain (16, 21, 35). However, unlike the *emm*75-ST150/657 isolates previously circulating in Portugal and the *emm*75-ST49 isolates identified in Spain and Houston (21, 35), which carry *mef*(A)-*msr*(D) in ɸ1207.3, the *emm*75-ST49 isolates from this study harbor a new ɸ1207.3 variant that likely emerged through recombination between ɸ1207.3 and other phages (Fig. 3). Further studies are necessary to determine if this variant encodes a functional and transferable phage.

Macrolide-resistant *emm*2 isolates have been infrequently isolated in Portugal and elsewhere (7, 9, 42). Despite the low genetic diversity of the national and international *emm*2 isolates, the closer relatedness of the resistant isolates in this study to international macrolide-susceptible isolates than to previous national resistant isolates is consistent with a recent introduction of an *emm*2-ST55 lineage carrying *mef*(A)-*msr*(D) in Portugal, possibly resulting from a novel acquisition of ɸ1207.3 by previously susceptible isolates. The two *mef*(A)-negative isolates genetically closer to our macrolide-resistant isolates (Fig. S2) were each temporally closer to its linked resistant isolate, despite being geographically distant (a US isolate from 2015 linked to a Portuguese 2019 resistant isolate and an Australian isolate from 1994 linked to a Portuguese 1998 resistant isolate), further supporting the occurrence of two independent phage acquisitions.

The main limitation of this study is that isolates were collected at a single hospital center, covering a limited geographic area. As a result, the findings may not fully represent the national antimicrobial resistance rates or genetic diversity and could be more influenced by any local outbreaks. In this regard, although *emm*2-ST55 isolates were detected from June to December 2019, continued surveillance across multiple hospitals is needed to determine whether the surge in this previously rare lineage was a regional event or part of a nationwide trend, and whether it persisted after the COVID-19 pandemic.

Our data showed that macrolide resistance is still low among streptococcal pharyngitis in Portugal. This occurs despite clonal shifts in macrolide-resistant lineages, often linked to changes in dominant macrolide resistance phenotypes and to resistance peaks. Moreover, macrolide resistance remains restricted to a few limited genetic lineages with marked clonal and epidemiological differences from macrolide-susceptible isolates.

## Data Availability

Raw sequencing data obtained in this study and sample metadata were deposited in the European Nucleotide Archive (ENA) under project accession number PRJEB88449. Sample accession numbers are provided in Table S1.

## AFTER EPUB

*[This article was published on 10 October 2025 with an error in the supplemental tables. The supplemental tables were corrected in the current version, posted on 17 October 2025.]*

## SUPPLEMENTAL MATERIAL

Supplemental material aac.00968-25-s0001.pdf Supplemental materials and methods; Fig. S1 to S9.

Supplemental tables aac.00968-25-s0002.xlsx Tables S1 and S2.

ASM does not own the copyrights to Supplemental Material that may be linked to, or accessed through, an article. The authors have granted ASM a non-exclusive, world-wide license to publish the Supplemental Material files. Please contact the corresponding author directly for reuse.
