# Supplementary material for: Increase of macrolide resistance among Streptococcus pyogenes pharyngitis driven by a mef(A)-msr(D)/emm2-ST55 lineage in Portugal (2014–2019)
Source: Antimicrob Agents Chemother. 2025 Oct 10;69(11):e00968-25. doi: 10.1128/aac.00968-25 (PMC12587576; doi:10.1128/aac.00968-25)
Supplement: Supplemental material — s and methods; Fig. S1 to S9. [file aac.00968-25-s0001.pdf]

Increase of macrolide resistance among *Streptococcus pyogenes* pharyngitis driven by a *mef*(A)-*msr*(D)/*emm*2-ST55 lineage in Portugal (2014-2019)

Supplemental Materials and Methods

High-throughput sequencing

Genomic DNA was extracted and purified from overnight cultures of GAS in Todd-Hewitt broth (Becton, Dickinson and Company, Sparks, MD, USA) with the PureLink™ genomic DNA minikit (Invitrogen, Carlsbad, CA, USA), adding 45 U of mutanolysin (Sigma-Aldrich, St. Louis, MO, USA) and 86 µg of hyaluronidase (Sigma-Aldrich, St. Louis, MO, USA) to the bacterial lysis step. WGS libraries were prepared with the Nextera DNA library preparation kit (Illumina, San Diego, CA, USA) and sequenced with an Illumina MiSeq or NextSeq instrument.

Sequencing data analysis

The sequencing data for the 391 pharyngeal GAS isolates recovered throughout Portugal were assembled with INNUca v4.2.3 (1) with the following parameters: --speciesExpected Streptococcus pyogenes, --genomeSizeExpectedMb 2, --runInsertSize, --maxNumberContigs 300, --trueCoverageProceed, and --fastQCproceed.

Genome annotation was performed with Prokka 1.14.6 (2) with the following parameters: --addgenes, --usegenus, --rfam, --rnammer, --increment 10, --mincontiglen 1, --gcode 11 and --kingdom Bacteria.

In silico Sequence Type (ST) prediction was performed using MLST v2.23.0 (3) with default parameters and the PubMLST database available at https://pubmlst.org/organisms/streptococcus-pyogenes/.

The *emm* type was determined from the draft genomes using emmtyper v0.2.0 (4) in verbose mode and the database available at https://www2.cdc.gov/vaccines/biotech/strepblast.asp.

The core-genome multilocus sequence typing profiles were determined with chewBBACA 3 (5) using default parameters and the *Streptococcus pyogenes* wgMLST schema available in the Chewie-NS platform (6). Allelic profiles of the core loci (shared by 100% of the isolates under analysis) were used to create minimum spanning trees with the goeBURST algorithm in the online version of PHYLOViZ (7).

The presence of antimicrobial resistance conferring genes was determined using ABRicate (8) with default parameters and the *ncbi* database.

For identification of the MGEs carrying macrolide resistance genes, the contigs carrying *erm*(A), *erm*(B), *erm*(T), or *mef*(A)-*msr*(D) genes were extracted from the assemblies and aligned with the sequences of known MGEs carrying the same combinations of antimicrobial resistance genes (9–12) using the ClustalW or progressive Mauve algorithms with default settings as implemented in Geneious R8 (Biomatters, Auckland, New Zealand). When necessary, blastn was used to compare complete or partial MGE sequences to the NCBI core nucleotide database. ICEfinder (13) was used to identify putative integrative conjugative elements (ICEs).

The figures comparing the mobile genetic elements were created with pyGenomeViz (14). A custom Python script was created to adjust the default BLASTn (15) parameters used by pyGenomeViz when aligning sequences in GenBank files. The following optional BLASTn parameters were used: -word_size 28 -gapopen 10 -gapextend 5 -penalty -3 -reward 1 -xdrop_ungap 1 -xdrop_gap 1 -xdrop_gap_final 10. These options increased alignment sensitivity, allowing to more accurately represent

sequence differences in the figures created by pyGenomeViz. Additionally, an option to adjust the offset of the feature tracks based on the leftmost alignment was implemented to align the feature tracks vertically.

Supplemental Figures

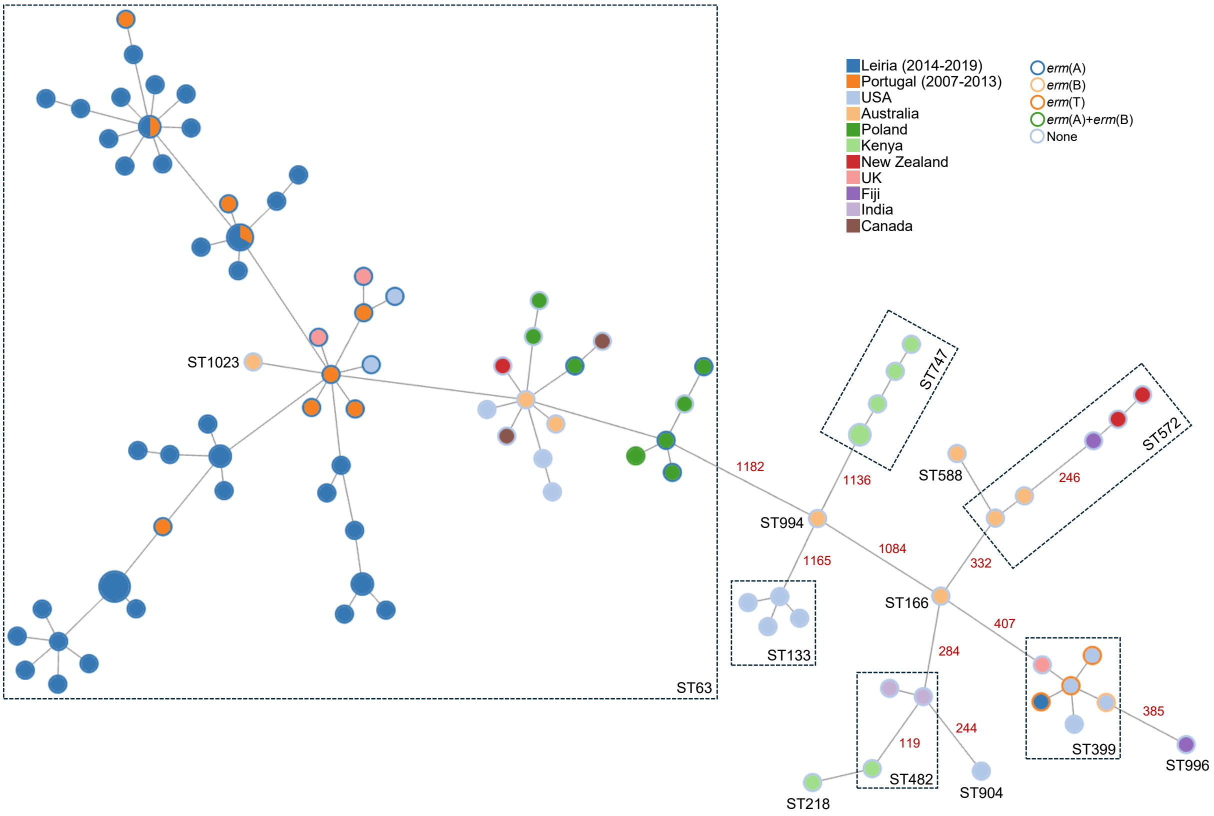

Fig. S1. Minimum spanning tree generated with the cgMLST profiles of *emm*77 GAS isolates. The tree includes: all macrolide-resistant pharyngeal *emm*77 isolates recovered at ULSRL, 2014-2019 (*n*=42) (this study), macrolide-resistant pharyngeal *emm*77 isolates recovered throughout Portugal, 2007-2013 (*n*=9) (16), complete genomes of *emm*77-ST63 isolates from Poland (*n*=8) (17), and diverse international *emm*77 isolates (*n*=41) (18). The size of each node is proportional to the number of isolates with that particular cgMLST profile. Nodes are colored according to geographic origin, with an outer ring colored according to the macrolide resistance genes identified in the isolates. Link distances >100 allelic differences are labeled in red (from a total of 1,314 compared loci). The dashed boxes group isolates sharing the same multilocus sequence type (ST).

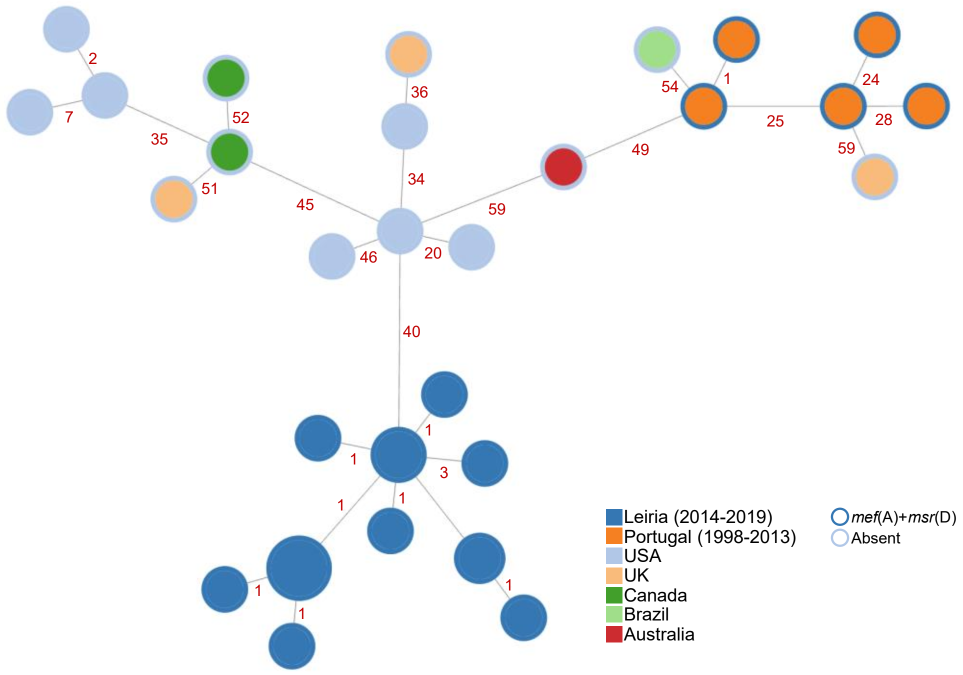

Fig. S2. Minimum spanning tree generated with the cgMLST profiles of *emm*2 GAS isolates. The tree includes: all macrolide-resistant pharyngeal *emm*2 isolates recovered at ULSRL, 2014-2019 (*n*=17) (this study), macrolide-resistant pharyngeal *emm*2 isolates recovered throughout Portugal, 1998-2013 (*n*=5) (16, 19), and diverse international *emm*2 isolates (*n*=14) (18). The size of each node is proportional to the number of isolates with that particular cgMLST profile. Nodes are colored according to geographic origin, with an outer ring colored according to the macrolide resistance genes identified in the isolates. Link distances are labeled in red as the number of allelic differences between nodes (from a total of 1,427 compared loci). All isolates share the same multilocus sequence type (ST55).

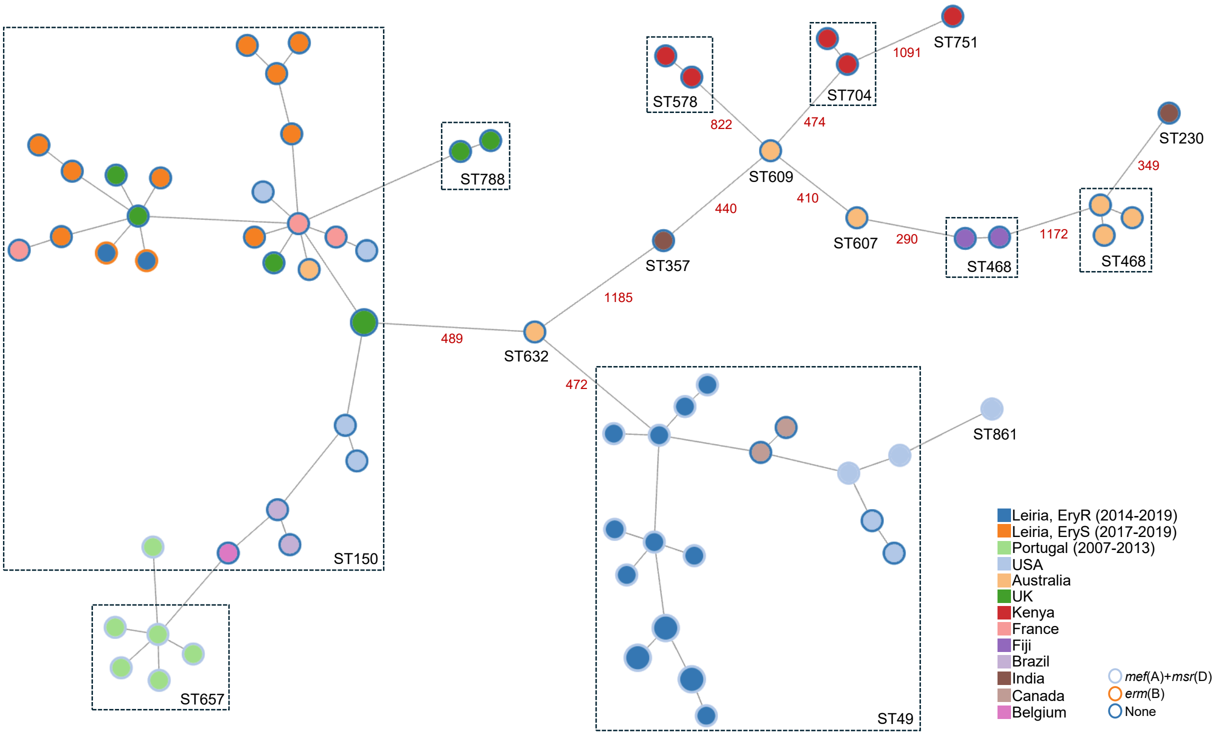

Fig. S3. Minimum spanning tree generated with the cgMLST profiles of *emm*75 GAS isolates. The tree includes: all macrolide-resistant pharyngeal *emm*75 isolates recovered at ULSRL, 2014-2019 (*n*=17) (this study), macrolide-susceptible pharyngeal *emm*75 isolates recovered in ULS Leiria, 2017-2019 (*n*=9) (this study), macrolide-resistant pharyngeal *emm*75 isolates recovered throughout Portugal, 2007-2013 (*n*=6) (16), and diverse international *emm*75 isolates (*n*=40) (18). The size of each node is proportional to the number of isolates with that particular cgMLST profile. Nodes are colored according to geographic origin, with an outer ring colored according to the macrolide resistance genes identified in the isolates. Link distances >100 allelic differences are labeled in red (from a total of 1,305 compared loci). The dashed boxes group isolates sharing the same multilocus sequence type (ST).

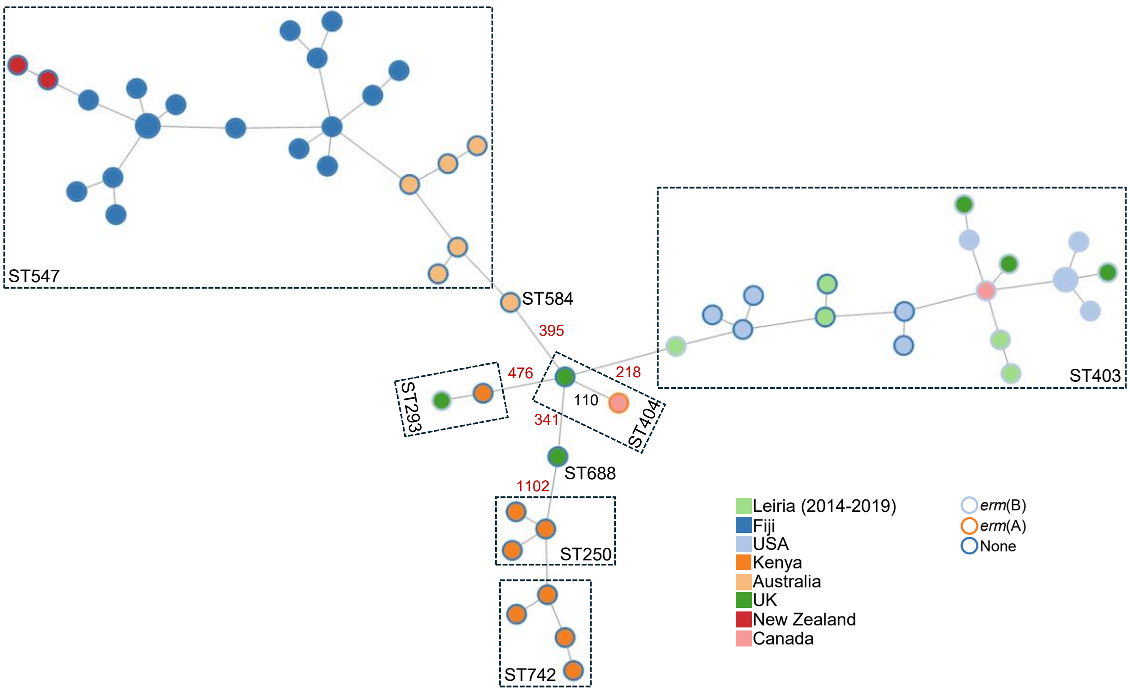

Fig. S4. Minimum spanning tree generated with the cgMLST profiles of *emm*11 GAS isolates. The tree includes: all macrolide-resistant pharyngeal *emm*11 isolates recovered at ULSRL, 2014-2019 (*n*=3) (this study), macrolide-susceptible pharyngeal *emm*11 isolates recovered in ULSRL, 2017-2019 (*n*=2) (this study), and diverse international *emm*11 isolates (*n*=51) (18). The size of each node is proportional to the number of isolates with that particular cgMLST profile. Nodes are colored according to geographic origin, with an outer ring colored according to the macrolide resistance genes identified in the isolates. Link distances >100 allelic differences are labeled in red (from a total of 1,342 compared loci). The dashed boxes group isolates sharing the same multilocus sequence type (ST).

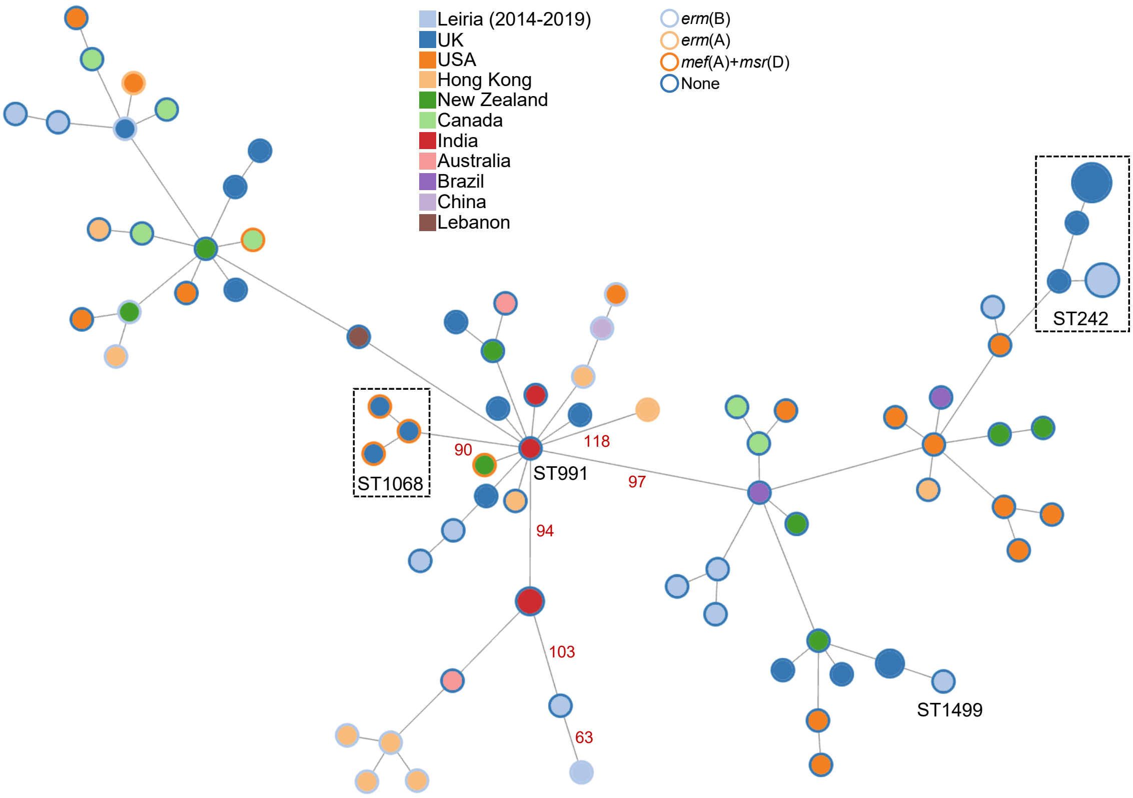

Fig. S5. Minimum spanning tree generated with the cgMLST profiles of *emm*12 GAS isolates. The tree includes: all macrolide-resistant pharyngeal *emm*12 isolates recovered at ULSRL, 2014-2019 (*n*=1) (this study), macrolide-susceptible pharyngeal *emm*12 isolates recovered in ULSRL, 2017-2019 (*n*=13) (this study), and diverse international *emm*12 isolates (*n*=69) (18). The size of each node is proportional to the number of isolates with that particular cgMLST profile. Nodes are colored according to geographic origin, with an outer ring colored according to the macrolide resistance genes identified in the isolates. Link distances >50 allelic differences are labeled in red (from a total of 1,334 compared loci). The dashed boxes group isolates sharing the same multilocus sequence type (ST). Unlabeled nodes shared ST36.

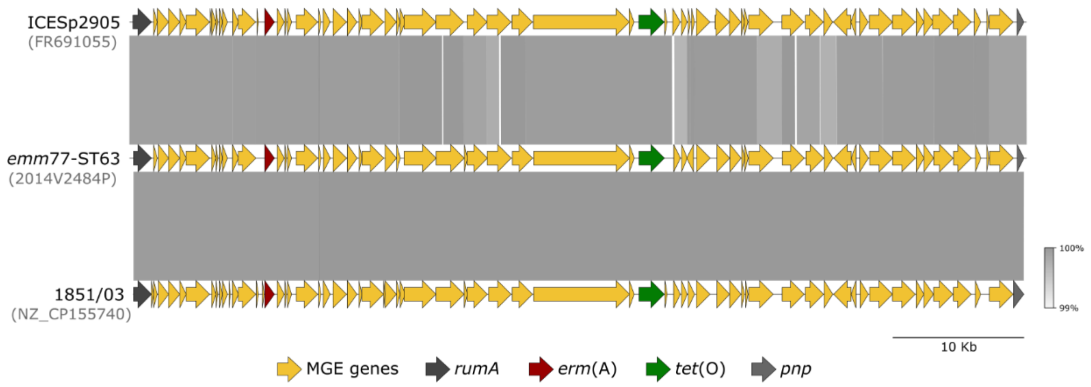

Fig. S6. Comparison of the element carrying the *erm*(A) and *tet*(O) genes in *emm*77-ST63 isolates from this study with the reference sequence of ICE*Sp2905* (20), and the *emm*77-ST63 strain 1851/03 from Poland (17). The name of the representative *emm*77-ST63 isolate used or the GenBank accession numbers of the sequences are indicated in gray. The gray-shaded areas connect regions based on nucleotide identity (99%-100%). Annotated coding sequences are represented as arrows: genes belonging to the mobile genetic element (MGE) in orange, except for *erm*(A) (dark red) and *tet*(O) (dark green), and the chromosomal genes (*rumA* and *pnp*) in shades of gray.

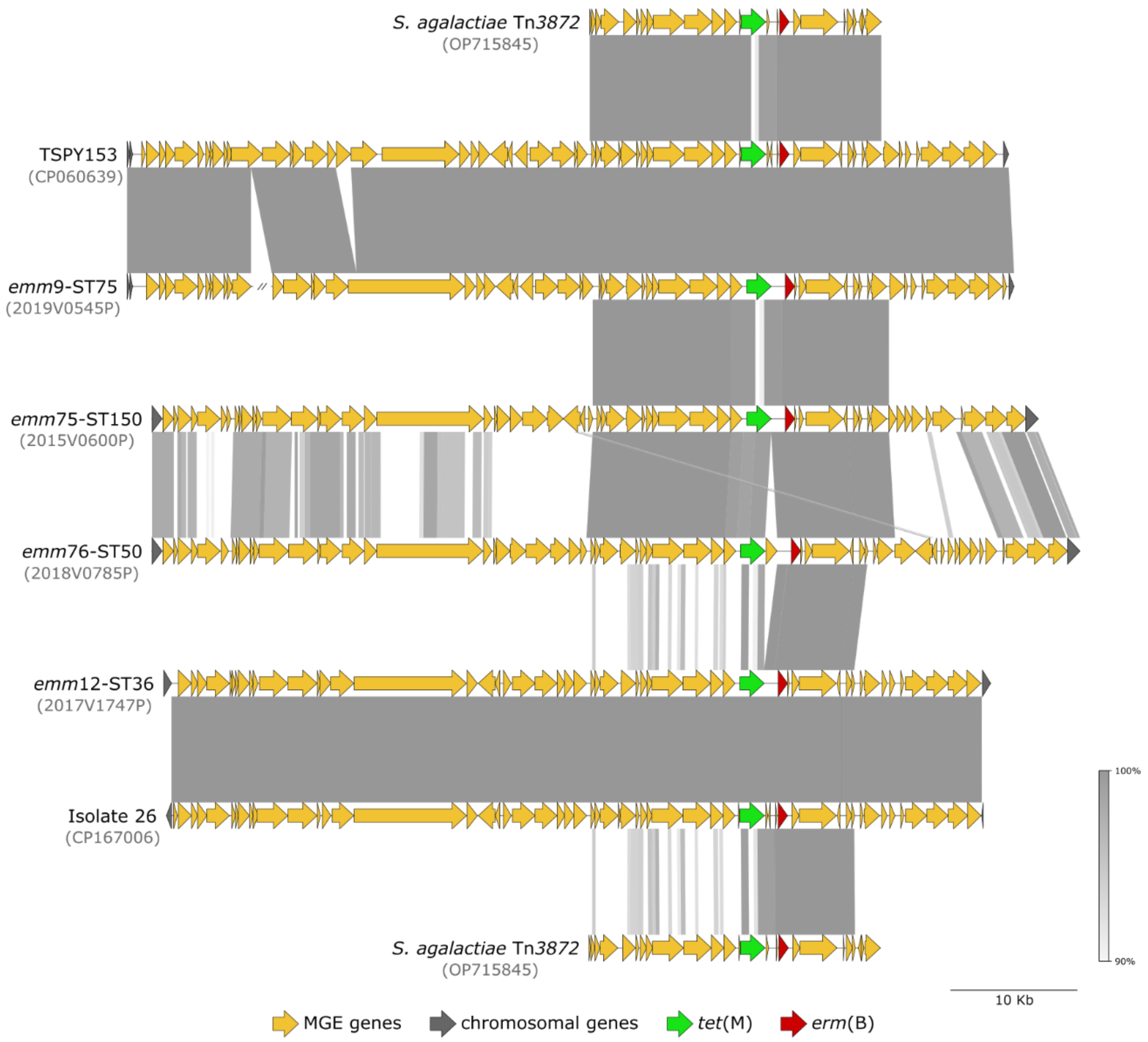

Fig. S7. Comparison of the Tn*3872*- and Tn*3872*-like-carrying elements identified in this study with the reference sequence of Tn*3872* from *Streptococcus agalactiae* and with the elements identified in *emm*9-ST75 isolate TSPY153 from Houston and in *emm*12-ST36 isolate 26 from Italy (21). The name of the representative isolates used for each lineage, or the GenBank accession numbers of the sequences are indicated in gray. The gray-shaded areas connect regions based on nucleotide identity (90%-100%). Annotated coding sequences are represented as arrows: genes belonging to the mobile genetic element (MGE) in orange, except for *erm*(B) (red) and *tet*(M) (green), and the chromosomal genes in gray. The double slash indicates sequence interruption due to assembly into different contigs.

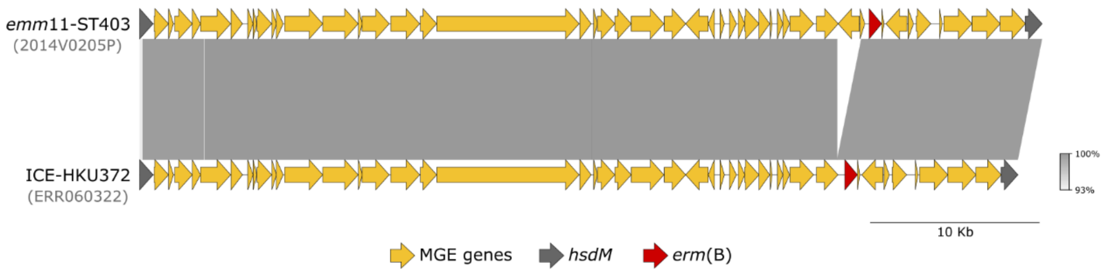

Fig. S8. Comparison of the element carrying the *erm*(B) gene in the *emm*11-ST403 isolate recovered in 2014 in this study with the sequence of ICE-HKU372 (10). The name of the *emm*77-ST63 isolate and the GenBank accession number of the ICE-HKU372-containing sequence are indicated in gray. The gray-shaded areas connect regions based on nucleotide identity (93%-100%). Annotated coding sequences are represented as arrows: genes belonging to the mobile genetic element (MGE) in orange, except for *erm*(B) (red), and the interrupted chromosomal *hsdM* gene in gray.

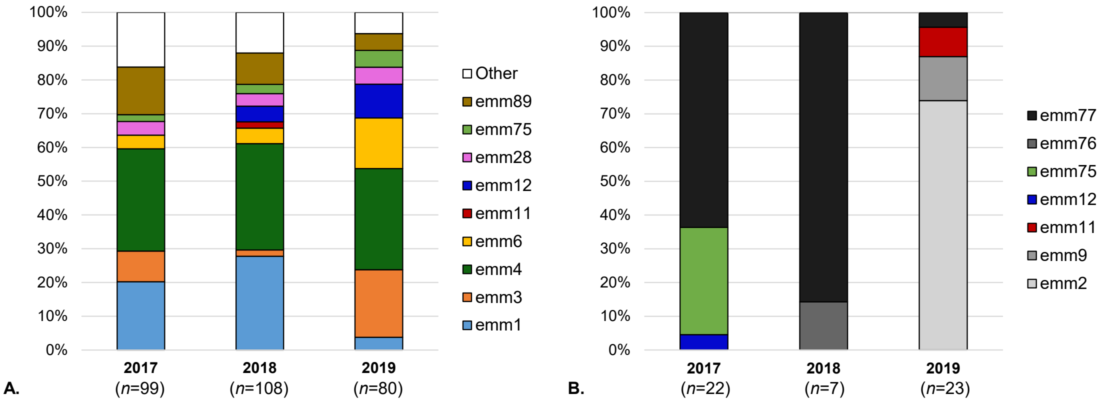

Fig. S9. Yearly *emm* type prevalence among 287 macrolide-susceptible (A) and 52 macrolide-resistant (B) pharyngeal GAS isolated between 1 January 2017 and 31 December 2019. "Other" includes *emm* types that comprised <10 isolates and that were not present among erythromycin-resistant isolates [*emm*22 (*n*=5), *emm*42 (*n*=1), *emm*44 (*n*=7), *emm*87 (*n*=7), and *emm*94 (*n*=8)], as well as non-typable isolates due to *emm* gene deletions or fusions (*n*=6).

References

1. Bioinformatics @ Molecular Microbiology and Infection Unit. 2020. INNUca. Python. https://github.com/B-UMMI/INNUca. Accessed 5 August 2024.

2. Seemann T. 2014. Prokka: rapid prokaryotic genome annotation. Bioinformatics 30:2068–2069.

3. Seemann T. 2024. tseemann/mlst. Shell. https://github.com/tseemann/mlst. Accessed 18 September 2024.

4. Microbiological Diagnostic Unit Public Health Laboratory. 2023. emmtyper - Emm Automatic Isolate Labeller (v0.2.0). Jupyter Notebook. https://github.com/MDU-PHL/emmtyper. Accessed 5 August 2024.

5. Silva M, Machado MP, Silva DN, Rossi M, Moran-Gilad J, Santos S, Ramirez M, Carriço JA. 2018. chewBBACA: A complete suite for gene-by-gene schema creation and strain identification. Microb Genom 4:e000166.

6. Mamede R, Vila-Cerqueira P, Silva M, Carriço JA, Ramirez M. 2021. Chewie Nomenclature Server (chewie-NS): a deployable nomenclature server for easy sharing of core and whole genome MLST schemas. Nucleic Acids Res 49:D660–D666.

7. Ribeiro-Gonçalves B, Francisco AP, Vaz C, Ramirez M, Carriço JA. 2016. PHYLOViZ Online: web-based tool for visualization, phylogenetic inference, analysis and sharing of minimum spanning trees. Nucleic Acids Res 44:W246-251.

8. Seemann T. 2020. ABRicate - Mass screening of contigs for antimicrobial and virulence genes. Perl. https://github.com/tseemann/abricate. Accessed 5 August 2024.

9. Berbel D, Càmara J, González-Díaz A, Cubero M, López de Egea G, Martí S, Tubau F, Domínguez MA, Ardanuy C. 2021. Deciphering mobile genetic elements disseminating macrolide resistance in *Streptococcus pyogenes* over a 21 year period in Barcelona, Spain. J Antimicrob Chemother 76:1991–2003.

10. Davies MR, Holden MT, Coupland P, Chen JHK, Venturini C, Barnett TC, Zakour NLB, Tse H, Dougan G, Yuen K-Y, Walker MJ. 2015. Emergence of scarlet fever *Streptococcus pyogenes emm*12 clones in Hong Kong is associated with toxin acquisition and multidrug resistance. Nat Genet 47:84–87.

11. Sanson MA, Macias OR, Shah BJ, Hanson B, Vega LA, Alamarat Z, Flores AR. 2019. Unexpected relationships between frequency of antimicrobial resistance, disease phenotype and *emm* type in group A *Streptococcus*. Microb Genom 5.

12. Varaldo PE, Montanari MP, Giovanetti E. 2009. Genetic elements responsible for erythromycin resistance in streptococci. Antimicrob Agents Chemother 53:343–353.

13. Microbial Bioinformatics Group at MML, SJTU. ICEfinder. https://bioinfo-mml.sjtu.edu.cn/ICEfinder/ICEfinder.html. Accessed 14 March 2025.

14. Shimoyama Y. 2024. pyGenomeViz: A genome visualization python package for comparative genomics. Python. https://github.com/moshi4/pyGenomeViz. Accessed 11 April 2025.

15. Camacho C, Coulouris G, Avagyan V, Ma N, Papadopoulos J, Bealer K, Madden TL. 2009. BLAST+: architecture and applications. BMC Bioinformatics 10:421.

16. Silva-Costa C, Ramirez M, Melo-Cristino J, Portuguese Group for Study of Streptococcal Infections. 2015. Declining macrolide resistance in *Streptococcus pyogenes* in Portugal (2007-13) was accompanied by continuous clonal changes. J Antimicrob Chemother 70:2729–2733.

17. Gawor J, Żuchniewicz K, Ojeda Saavedra M, Beres SB, Kiedrowska M, Wróbel-Pawelczyk I, Kozińska A, Gromadka R, Musser JM, Sitkiewicz I, Kern-Zdanowicz I. 2024. ICE*Sp1109*, a novel hybrid Integrative Conjugative Element of macrolide-resistant *Streptococcus pyogenes* serotype M77 collected between 2003 and 2017 in Poland. J Infect Dis jiae473.

18. Davies MR, McIntyre L, Mutreja A, Lacey JA, Lees JA, Towers RJ, Duchêne S, Smeesters PR, Frost HR, Price DJ, Holden MTG, David S, Giffard PM, Worthing KA, Seale AC, Berkley JA, Harris SR, Rivera-Hernandez T, Berking O, Cork AJ, Torres RSLA, Lithgow T, Strugnell RA, Bergmann R, Nitsche-Schmitz P, Chhatwal GS, Bentley SD, Fraser JD, Moreland NJ, Carapetis JR, Steer AC, Parkhill J, Saul A, Williamson DA, Currie BJ, Tong SYC, Dougan G, Walker MJ. 2019. Atlas of group A streptococcal vaccine candidates compiled using large-scale comparative genomics. Nat Genet 51:1035–1043.

19. Silva-Costa C, Ramirez M, Melo-Cristino J. 2006. Identification of macrolide-resistant clones of *Streptococcus pyogenes* in Portugal. Clin Microbiol Infect 12:513–518.

20. Brenciani A, Tiberi E, Bacciaglia A, Petrelli D, Varaldo PE, Giovanetti E. 2011. Two distinct genetic elements are responsible for *erm*(TR)-mediated erythromycin resistance in tetracycline-susceptible and tetracycline-resistant strains of *Streptococcus pyogenes*. Antimicrob Agents Chemother 55:2106–2112.

21. Arcari G, Novazzi F, Colombini L, Drago Ferrante F, Boutahar S, Paolo Genoni A, Cassani G, Gigante P, Carbotti M, Bianco A, Tirziu M, Capuano R, Pasciuta R, Iannelli F, Clementi N, Santoro F, Mancini N. 2025. Post-pandemic upsurge in Group A *Streptococcus* infections at an Italian tertiary university hospital. Microbiol Spectr 13:e0249424.
